# Logistics integration in the supply chain: a resource dependence theory perspective

**DOI:** 10.1186/s40887-020-00039-w

**Published:** 2020-10-14

**Authors:** Sung Tae Kim, Hong-Hee Lee, Taewon Hwang

**Affiliations:** 1 Greehey School of Business, St. Mary’s University, 1 Camino Santa Maria, San Antonio, TX 78228 USA; 2 College of Business Administration, Valdosta State University, 1500 N. Patterson St, Valdosta, GA 31698 USA

**Keywords:** Logistics integration, Supply chain performance, Trust, Satisfaction, Commitment

## Abstract

Firms have strategically used cooperative linkages to establish competitiveness. In this study, we incorporated the resource dependency theory view to assess how trust, satisfaction, and commitment affect firms’ decisions on logistics integration. Also, we examined the link between logistics integration and supply chain performance. The study collected data from 250 South Korean manufacturers for analysis. The results revealed positive impacts of trust, satisfaction, and commitment on logistics integration between manufacturing firms and logistics service providers that enhances logistics service capabilities of the firms. Furthermore, our study showed that building a strategic relationship for logistics services helps the manufacturing firms improve their business and operations performances in their supply chain. Implications and suggestions for future research are discussed.

## Introduction

Globalization has brought fundamental changes to the business environment. In response to this change, an increasing number of firms have been seeking to develop strong relationships with their business partners because it is often difficult for a single firm to obtain all the resources required to tackle rapidly changing business environments [1]. Many manufacturers have adopted strategic alliances with their supply chain members to take advantage of the economies of scale [2]. They have supply chain participants involved with sharing information, knowledge, resources, and competencies—an approach to strengthen the overall competitive position of the whole supply chain [3]. Especially, it was found that close relationships between key supply chain members and logistics service providers (LSPs) positively influence logistics and distribution performance in the downstream, which in turn lead to better supply chain performance.

Hyundai Motors, a multinational automobile company, is a good example of a firm which established a successful supply chain partnership. It has the world’s largest integrated automobile manufacturing facility in Ulsan, South Korea. This facility has an annual production capacity of 1.6 million units [4]. The company’s corporate headquarter in Seoul is responsible for managing supply chains both domestically and globally. One of the challenges of supply chain management (SCM) is to develop efficient and effective supply networks for local suppliers so that they can utilize advanced infrastructure and logistics services. Another challenge is to obtain a competitive logistics because the company needs to send a substantial amount of parts and components to global plants for reassembly in locations widely spread throughout the world such as Europe, South and North America, China, and India. To overcome these challenges, the company has made moves toward vertical integration with Glovis, one of the largest LSPs in South Korea.

Previous studies highlighted the importance of synchronized logistical activities among supply chain members. Lai et al. [5] contributed to understanding the three key factors (trust, satisfaction, and commitment) for supporting effective logistics integration. They investigated how each factor is connected to logistics integration as well as firm’s financial performance. This study attempts to extend the results of their study by investigating the impact of the three factors on logistics integration and supply chain performance in the context of logistics outsourcing.

This paper is organized as follows. The next section provides the theoretical background of the study and develops hypotheses. Then, research methods are described including data collection, followed by reporting analysis results and discussion. Lastly, we conclude the paper by discussing the implications of the study results, including limitations of the study and future research needs.

## Literature review and hypotheses development

### Logistics integration

In the logistics and SCM context, the term logistics integration can be defined as the degree to which a client firm strategically collaborates with its LSP to manage its intra- and inter-organization processes [6]. In a network-based business environment, firms place a great level of strategic importance on logistics integration [7]. Chang and Ku [8] pointed out that logistics integration is now an umbrella term that encompasses a wide range of inter-functional activities between the logistics and marketing department, IT department, and so on. Highly integrated logistics processes involve dynamically coordinated business processes both within and outside the organizational boundaries [9].

The role of logistics functions in the enterprise has considerably changed over the years. Logistics integration used to be a vague concept. Until the 1970s, logistics operations were primarily carried out in-house and often seen as a cost center with little capacity for differentiation. This traditional perspective changed in the 1980s as firms began to outsource their logistics activities to LSPs, which support a client firm’s supply chain operations such as procurement, inventory control, warehousing, and transportation [10]. This new outsourcing practice is largely the results of treating LSPs as strategic partners in improving supply chain performance [11, 12]. Such a perspective has emphasized that logistics integration goes beyond simple information sharing between participants involved in the supply chain relationship. Logistics outsourcing has now become common as more firms become aware of the advantages that LSPs offer. Today’s LSPs can help client firms move beyond mere cost reduction to more strategic, value-creating activities along the supply chain [13].

### Logistics trust

Drawing from resource dependence theory (RDT), this study identifies three antecedents of logistics integration, namely, trust, commitment, and satisfaction. RDT has been widely used to explain why more and more firms are entering into inter-organizational arrangements [14]. In contemporary business environments, it is often difficult for a single firm to possess all resources required to achieve a sustainable competitive advantage [15]. By forming alliances and joint ventures, a firm might gain effective access to the knowledge and resources of business partners [16]. In other words, firms that lack specific resources may be able to acquire these resources by establishing external relationships. RDT suggests that firms become dependent upon each other in order to create such complementary assets [17]. Researchers found that there has been a strong trend toward the development of core capabilities through knowledge exchange, investments in relation-specific assets, and complementary capabilities [18]. By recombining unique and inimitable resources, firms can improve their opportunities to successfully launch new products and services.

Following RDT, this study posits that three relationship factors, including trust, satisfaction, and commitment, are positively associated with logistics integration. First, trust generally refers to the willingness to depend on a party when one is confident in the actions of that party [19]. Trust exists when one party has confidence in the reliability and integrity of the other party [20]. Trust brings about a feeling of security, reduces uncertainty, and creates a supportive environment. Trust is the belief of a firm that its business partners will make sure that all actions will result in positive achievements for the firm [16]. Trust is one of the most commonly addressed factors of inter-organizational relationships [21]. In logistics outsourcing context, trust refers to the willingness of a client firm to depend on its LSP in whom it has confidence in creditability, competence, and benevolence [22]. This study argues that a client firm with a high level of trust in the LSP relationship is more likely to integrate the LSP’s service offerings into its logistics processes. These observations lead us to propose the following hypothesis:

*H1*. A client firm’s trust in its LSP is positively related to logistics integration.

### Logistics satisfaction

Satisfaction is another factor extensively examined in various business contexts over the last two decades [23]. Satisfaction in an inter-organizational relationship generally refers to the buyer’s attitude formed based on the experience with the supplier [24]. Positive affective states (e.g., greater satisfaction) are likely to strengthen feelings of safety, security, comfort, and confidence [25]. In the logistics outsourcing context, satisfaction refers to the degree to which a client firm is satisfied with its LSP [22]. The more satisfied the customer firm is with the previous LSP’s service, the stronger the integration between the two companies is expected. Wilson and Jantrania [26] identified satisfaction as a key element in constructing relationships among enterprises. In addition, Storbacka et al. [27] included satisfaction and communication as factors to build a high-quality relationship. This study argues that a client firm highly satisfied with the LSP relationship is more likely to integrate the LSP’s service offerings into its logistics processes. These observations lead us to suggest the following hypothesis:

*H2*. A client firm’s satisfaction with its LSPs is positively related to logistics integration.

### Logistics commitment

Commitment generally refers to the belief that a relationship is important that it warrants the maximum efforts to maintain it [28]. Commitment is an essential element in constructing successful long-term working relationships [29]. Commitment is significantly and positively related to business partners’ attitude toward the development of a sustainable supply chain relationship [30]. In logistics outsourcing context, commitment is an important factor in determining the effectiveness of LSP relationships [31]. When an LSP displays a higher level of commitment, its client firm is likely to have a stronger intention to continue the relationship with that LSP [32]. An LSP committed to understanding each customer’s unique needs has the ability to achieve a high-level integration across multiple supply chain partners [13]. This study argues that a client firm perceiving a strong commitment to the LSP relationship is more likely to integrate the LSP’s service offerings into its logistics processes. These observations lead us to propose the following hypothesis:

*H3*. A client firm’s perception on the commitment of its LSP is positively related to logistics integration.

### Supply chain performance

**Fig. 1:**
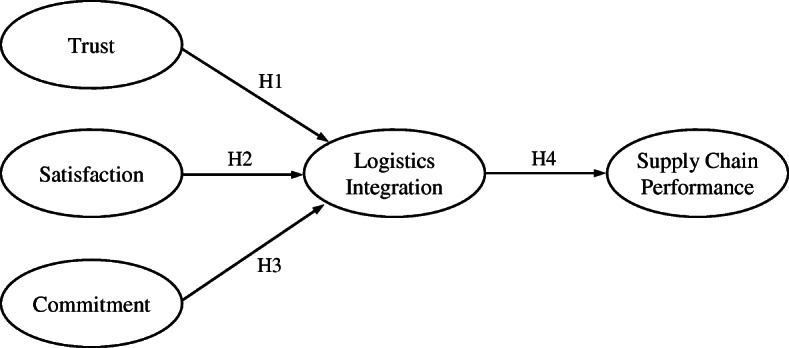
Research model

Logistics integration provides an LSP the opportunity to serve as an integral part of the supply chain rather than a separate entity [33]. Through logistics integration, a client firm can better understand each supply chain member’s point of view, share valuable information, and achieve collective goals. A client firm can effectively address all different requirements, expectations, and preferences along all stages of the supply chain [34]. The integration of logistics activities across organizational boundaries helps a client firm reduce supply chain uncertainties caused by a lack of information and knowledge [35]. A client firm working with an LSP can improve information processing capabilities by taking advantage of a huge amount of data generated along the supply chain [36]. In other words, the integration of logistics activities across organizational boundaries helps a client firm reduce inefficiencies involved in planning, manufacturing, and distribution activities [37]. Effective logistics integration has the potential to overcome supply chain risks (e.g., excess inventories, rush deliveries, and long lead times) [38]. In this way, logistics integration leads to a well-coordinated supply chain, promoting mutual benefits (e.g., large market share, operational efficiency, effective governance, and a satisfactory amount of profit) [39]. Thus, logistics integration can be considered a key factor for enhancing supply chain performance. These observations lead us to propose the following hypothesis:

*H4*. Logistics integration is positively related to supply chain performance. Figure 1 shows our research model containing the four hypotheses.

## Research methodology

### Questionnaire development

Lai et al. [5] tested the dependence in logistics outsourcing relationships. Main constructs used in the research were “trust,” “commitment,” “satisfaction,” and “logistics integration”. This paper adopted the measurement items of the first three dimensions and also added items from Chang and Ku [8] with modifications. Measurement items for “logistics integration” are adopted from Chang and Ku [8], Prajogo and Olhager [9], and Lai et al. [5]. This study utilized additional items that are found in other studies [40–44]. For the measurement items, a five-point Likert scale was used (1: strongly disagree to 5: strongly agree).

In developing the questionnaire, the double translation protocol was used. The questionnaire was developed in English first and then was translated into Korean. After the translation, the questionnaire was presented to a panel of experts from both academia and SCM practitioners to solicit their feedback regarding the survey items. To assure translation equivalence, the questionnaire translated into Korean was back-translated into English. The two English versions did not have any major difference. The scales used to measure this study’s constructs were developed based on an in-depth literature review, and existing scales were used wherever possible. Minor wording changes were made in order to adapt the scale to the specific supply chain management context. The measurement scales and their sources are shown in Table 2.

### Sampling and data collection

The data for this study was collected from Korean manufacturing firms. A mailing list of logistics or SCM departments was compiled from the list of partner companies of the Korea Trade-Investment Promotion Agency (KOTRA), and the survey was conducted in cooperation with a research-consulting firm. Approximately 1000 companies were randomly selected from the list. Senior or middle managers with direct responsibility for logistics or SCM were regarded as our target respondents. The survey team of the consulting firm first called the logistics or SCM department of the selected companies for their cooperation, and then, the questionnaire was sent to 350 companies that were willing to participate in the survey (See Table 1). A total of 250 responses were received. If any omitted questions were found, the survey team called the manager to complete the questionnaire.

**Table 1:** Characteristics of responding firms

	Frequency	%
A. Respondents’ job title		
Employee in charge	42	16.8
Middle manager	148	59.2
Senior executive	34	13.6
Top executive	26	10.4
Total	250	100.0
B. Respondents’ work experience (years)		
Less than 5	53	21.2
5–10	64	25.6
11–15	109	43.6
More than 15	24	9.6
Total	250	100.0
C. Firm size (no. of employees)		
Less than 100	52	20.8
100–400	67	26.8
401–700	72	28.8
701–1000	40	16.0
More than 1000	19	7.6
Total	250	100.0
D. Industry classification		
Steel parts	40	16.0
Electronics	83	33.2
Furniture	47	18.8
Plastic products	51	20.4
Others	29	11.6
Total	250	100.0

### Non-response bias analysis

A test for the non-response bias was conducted by comparing the early and late respondents. Responses received before the reminder email was regarded as early responses and those received after as late. *T* tests were conducted to check for differences between the two groups of respondents on important measures. There were no mean differences between the two sets on key attributes such as firm size.

## Results

### Respondent profile

Table 1 presents the general industry characteristics of the respondents. The responding companies represented largely 5 industries including electronics (83), plastic products (51), furniture (47), steel parts (40), and others (29). The participating firms were mostly small and medium-sized enterprises (SMEs), and the median firm size (in the number of employees) was 400. Respondents’ job titles ranged from the employee in charge of SCM to senior manager. Middle and senior managers and top executives represented more than 80% of the sample, and majority of the job titles were managers in charge of SCM. This result indicates that SCM of Korean small- and medium-sized manufacturers is under the supervision of higher-level managers with a minimum 7-year-experience in the industry.

### Analysis of reliability and validity

Table 2 shows constructs and survey items adopted for this study. The acceptability of the measurement model was examined by analyzing the convergence validity, discriminant validity, and reliabilities of all constructs. Convergent validity signifies that a set of measurement items represents one and the same underlying construct [45]. It was examined in two ways. We first assessed composite reliability (CR) scores for all constructs, and then, second, calculated the average variance extracted (AVE). As shown in Table 3, all constructs exceeded 0.7, the threshold of composite reliabilities, and all AVE estimates of the five constructs were greater than the cutoff point, 0.5 [46]. In conclusion, CR and AVE values provided strong support for convergent validity.

**Table 2:** Constructs and survey items

Constructs	Survey Items	References
**Trust**		
TR1	We can rely upon our logistics service providers’ promises.	Chang and Ku [8]; Lai et al. [5]
TR2	We respect our logistics service providers’ advice.	
TR3	We expect our logistics service providers’ behavior to be consistent with past behavior.	
TR4	Our logistics service providers expect us to maintain a close relationship with them.	
TR5	Our logistics service providers are sincere.	
**Satisfaction**		
SA1	We are pleased with the relationship of our logistics service providers.	Chang and Ku [8]; Lai et al. [5]
SA2	We have a favorable opinion on our logistics service providers’ performance.	
SA3	It is easy to do business with logistics service providers.	
SA4	Our logistics service providers have met our expectations on the support and services.	
**Commitment**		
CM1	Our logistics service providers have provided us with the help we need.	Chang and Ku [8]; Lai et al. [5]
CM2	Our logistics service providers have a standardized business process to help solve our problems.	
CM3	Our logistics service providers have treated us sincerely.	
CM4	Our logistics service providers have accurately provided customer services in agreement with the contract.	
**Logistics integration**		
LI1	We help our major logistics service provider improve its processes to better meet our needs.	Chang and Ku [8]; Prajogo and Olhager [9]; Lai et al. [5]
LI2	We hold meetings with our major logistics service provider on a regular basis to solve problems.	
LI3	We and our major logistics service provider work together as a team.	
LI4	We conduct joint planning with our major logistics service provider to resolve operational problems.	
LI5	We have developed a mutual understanding of responsibilities with our major logistics service provider.	
LI6	We make joint decisions with our major logistics service provider about ways to improve cost efficiency.	
LI7	We and our major logistics service provider jointly design customized order processes.	
**Supply chain performance**		
SP1	Our inventory cost is lowered.	Cheng and Tang [42]; Yang et al. [43]; Carr [44];
SP2	Return on assets has increased.	
SP3	Our suppliers’ product quality has improved.	
SP4	Our cost control has improved.	
SP5	Our suppliers’ cost control has improved.	
SP6	Market share has increased.	
SP7	Our main customers are satisfied with our logistics services.

**Table 3:** Reliability (composite reliability and AVEs) and correlations among latent variables

Construct	Composite Reliability	AVE	TR	SA	CM	LI	SP
Trust (TR)	0.914	0.684	**0.827**				
Satisfaction (SA)	0.823	0.544	0.522	**0.738**			
Commitment (CM)	0.920	0.743	0.581	0.492	**0.862**		
Logistics integration (LI)	0.922	0.635	0.471	0.466	0.412	**0.797**	
Supply chain performance (SP)	0.963	0.790	0.588	0.393	0.499	0.551	**0.889**

The squared correlation coefficients between two latent constructs to their AVE estimates were also compared [46]. According to this test, discriminant validity exists if the items share more common variance with their respective construct than any variance the construct shares with other constructs. Thus, the squared correlation coefficient between each pair of constructs should be less than the AVE estimates for each individual construct. Comparing the correlation coefficients with the AVE estimates reported in Table 3, all of the squared correlations were smaller than the AVE for each individual construct. Therefore, these results collectively provided evidence of discriminant validity among the theoretical constructs.

Reliability estimation was left for last because in the absence of a valid construct, reliability would not be meaningful [47]. Item-total correlation analysis results provided in Table 3 suggest a reasonable fit of the latent factors to the data collected. Cronbach’s α values for all factors were greater than 0.8, as shown in Table 4, which exhibit the internal consistency and validity of the constructs as they were well above the suggested lower limit of 0.7 [48]. This result provides support for high degrees of construct reliability. Table 5 shows cross-loading among the variables.

**Table 4:** Convergent validity (item loading)

Factors	Item no.	Standardized loading	*t* value	Cronbach’s α
Trust	TR1	0.743	23.904	0.915
	TR2	0.684	18.567	
	TR3	0.833	36.499	
	TR4	0.920	67.685	
	TR5	0.928	71.474	
Satisfaction	SA1	0.821	27.986	0.859
	SA2	0.789	24.717	
	SA3	0.722	19.475	
	SA4	0.786	24.824	
Commitment	CM1	0.791	29.753	0.920
	CM2	0.877	47.469	
	CM3	0.883	50.176	
	CM4	0.892	52.608	
Logistics integration	LI1	0.788	28.545	0.915
	LI2	0.797	29.892	
	LI3	0.861	43.600	
	LI4	0.841	38.129	
	LI5	0.809	32.533	
	LI6	0.748	23.840	
	LI7	0.601	13.954	
Supply chain performance	SP1	0.892	62.659	0.962
	SP2	0.908	72.926	
	SP3	0.869	52.624	
	SP4	0.935	103.313	
	SP5	0.950	127.461	
	SP6	0.952	132.391	
	SP7	0.687	20.069

**Table 5:** Cross-loading among variables

	TR	SA	CM	LI	SP
TR1	1.000	.360	.401	.256	.412
TR2	.610	.345	.461	.246	.355
TR3	.675	.383	.464	.354	.486
TR4	.661	.318	.427	.294	.498
TR5	.654	.350	.399	.321	.500
SA1	.360	1.000	.311	.269	.302
SA2	.357	.733	.293	.298	.285
SA3	.413	.643	.388	.325	.373
SA4	.463	.633	.340	.358	.350
CM1	.401	.311	1.000	.236	.426
CM2	.461	.324	.712	.295	.431
CM3	.413	.327	.680	.259	.395
CM4	.432	.344	.705	.246	.359
LI1	.256	.269	.236	1.000	.348
LI2	.243	.278	.249	.783	.337
LI3	.304	.258	.280	.675	.443
LI4	.341	.309	.391	.707	.430
LI5	.285	.221	.186	.696	.402
LI6	.361	.323	.430	.674	.571
LI7	.385	.290	.292	.790	.370
LQ1	.290	.277	.244	.453	.480
LQ2	.362	.250	.250	.350	.591
LQ3	.232	.271	.238	.460	.502
LQ4	.313	.291	.244	.443	.503
LQ5	.175	.305	.277	.425	.372
SP1	.412	.302	.426	.348	1.000
SP2	.433	.301	.408	.362	.903
SP3	.441	.299	.448	.313	.796
SP4	.415	.289	.378	.323	.833
SP5	.422	.244	.365	.364	.813
SP6	.440	.263	.398	.339	.828
SP7	.378	.308	.375	.289	.605

### Hypotheses testing

**Fig. 2:**
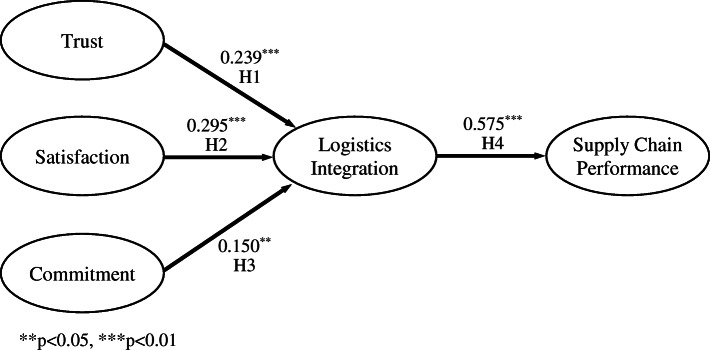
Hypothesized research model results

The results of the structural model are shown in Table 6. All fit indices were indicative of a decent fitting model. Figure 2 indicates support for all three preceding constructs on logistics integration (Hypotheses 1, 2, and 3): trust (0.229; *p* < 0.01), satisfaction (0.281; *p* < 0.01), and commitment (0.163; *p* < 0.05). The test results further supported Hypotheses 4 and 5 with significant positive correlation coefficients: logistics integration on logistics service quality (0.612; *p* < 0.01) and logistics integration on supply chain performance (0.293; *p* < 0.01). It is also found that logistics service quality on supply chain performance has a high correlation (0.405; *p* < 0.01).

**Table 6:** Results of hypotheses tests

	Path (from-to)	Path coefficient (*t* value)	Test results
H1	Trust ➔ logistics integration	0.239 (3.059)^***^	**Supported**
H2	Satisfaction ➔ logistics integration	0.295 (3.801)^***^	**Supported**
H3	Commitment ➔ logistics integration	0.150 (1.895) ^**^	**Supported**
H4	Logistics integration ➔ SC performance	0.575 (12.479)^***^	**Supported**

Fit indices: *χ*^2^ = 813.888 (d.f. = 316), χ^2^/d.f. = 2.58, CFI = 0.920, RMSEA = 0.079, SRMR = 0.088

^**^p < 0.05; ^***^p < 0.01

## Discussion

Based on resource dependence theory (RDT), we empirically examine the role of relational mechanisms in explaining the supply chain performance of firms in logistics outsourcing relationships. Congruous with our theoretical model, the results show that three relational factors, including trust, satisfaction, and commitment, are positively correlated with the logistics integration between client firms and their LSPs. Our findings also suggested that clients’ trust enhanced by the ability of logistics firms that perform and maintain expected services fosters the development of common goals and joint planning for logistics integration.

In addition, our findings showed that clients’ satisfaction positively influences logistics service performance in terms of meeting client’s expectations or performing flexible logistics operation. This result highlights that a high level of logistics service satisfaction facilitates logistics integration to maintain responsive and responsible operations to better deal with uncertainty in supply chains.

Similarly, the increasing environmental uncertainty poses significant challenges for firms that seek business partners that can help respond more effectively to rapidly changing markets. Our findings suggest that the commitment of a logistics firm to solve a company’s logistics-related issues increases the willingness of the customer to formulate a strategic integration with the logistics firm.

Lastly, our study demonstrated that supply chain performance is significantly associated with logistics integration which has emerged as a dominant competency in regard to customizing order processes, improving material flows, improving cost efficiency, and refining overall value stream. This finding is consistent with previous studies that asserted the key to constructing a highly competitive supply chain is by implementing end-to-end logistics solutions [9, 49, 50]. Such a strategic integration also helps continuously match logistics service capabilities with rising customer expectations and thus helps client firms improve product quality as well as market share.

## Implications and future research

Our study contributes to the literature in several important aspects. First, although the RDT-based mechanism has been investigated in the literature, we identify inter-organizational factors including trust, satisfaction, and commitment which can foster client firms’ willingness to integrate their logistics and supply chains. This is one of a few studies that build and empirically validated an integrated theoretical framework incorporating all three factors. This examination leads us to extend the logistics integration and logistics service quality literature by providing a more comprehensive view on the value of firms’ attempts to achieve logistics integration across global supply chains.

Second, in line with RDT, partner collaboration is a key for a firm to resolve operational difficulties. It is reasonable for firms to join in partnerships when they can sense strategic symbiotic relationships with one another or they can envision the complementary role of their operations and resources. Moreover, partner collaboration mitigates the risks of unexpected supply chain disruptions as shown by the recent COVID-19 pandemic. Most businesses are operating under a high level of environmental uncertainty. In such uncertain environments, firms need to eliminate unpredictable factors, which may lead to various negative consequences for them. Our results suggest that inter-organizational trust and customer’s satisfaction on logistics service are identified as the two factors relatively more critical to the successful implementation of logistics integration.

Third, our study provides important empirical support for a robust measurement model. The measures can be used to further test the relationships between logistics integration and logistics service performance. Moreover, our investigation reinforces the measurement model by considering perfect mediation effects in the model. Our statistical analyses show that none of the three factors had a direct effect on supply chain performance at the 5% significance level. In other words, trust, satisfaction, and commitment are mediated by logistics integration for the companies to obtain significant supply chain performance outcomes. This finding strengthens the importance of building high levels of trust, satisfaction, and commitment among supply chain members. Furthermore, the measurement model was originally developed and tested in China [5]. The contribution of this examination is significant in that it is one of a few empirical studies which used the measurement items and scales for estimating factors for logistics integration and supply chain performance among Korean enterprises and attempted to refine the measurement items with modification.

The study results have significant implications in the current pandemic crisis as well. Disruption has come to the global supply chain environment, and many companies have suffered from the impact of the pandemic, and some of their activities are minimized or completely stopped. Many manufacturers are seriously impacted by the interruptions of globalization occurred by the crisis [51]. Even crucial operations in businesses are experiencing major disruptions as suppliers experience difficulties with production [52]. The relationship formation between LSPs and their client firms is becoming more important in these unprecedented times. LSPs are more required to be more closely integrated with their client firms than ever before, and in order to do so, they need to constantly satisfy their client firms and build trust.

Tightly integrated LSPs with client firms might get back to normal more easily. Businesses are trying to restart their operations after the pandemic crisis in many ways. It takes a great deal of time, money, and effort to restart operations. To arrange the operations to restart, they need to (1) define capacities needed to start again, not just available ones; (2) assess the level of commitment of resources; and (3) contribute to the supply chain alignment [52]. Flexibility and ease of such tasks seem to be dependent on logistics integration. Restarting operations in the wake of the pandemic is already a big challenge even if you have good relationships with your partners without needing to suspect the motives of one another [53].

Recent years have seen a growing movement toward a view of relationship quality as a multi-faceted phenomenon [54]. This perspective is empirically supported by many literature reviews on relationship quality (e.g., Athanasopoulou [55] and Osobajo and Moore [56]). These reviews found an increasing tendency to treat relationship quality as an important strategic construct. For future research, it would be intriguing to apply the same approach employed in this study to other cultures or economies. Also, a longitudinal study will shed additional information for the long-term effects of the three key factors we examined in this study.

## Data Availability

The data for this study was collected from Korean manufacturing firms.

## Abbreviations

SCM: Supply chain management
LSPs: Logistics service providers
